# Alterations of PINK1-PRKN signaling in mice during normal aging

**DOI:** 10.1080/27694127.2024.2434379

**Published:** 2024-12-07

**Authors:** Zahra Baninameh, Jens O. Watzlawik, Xu Hou, Tyrique Richardson, Nicholas W. Kurchaba, Tingxiang Yan, Damian N. Di Florio, DeLisa Fairweather, Lu Kang, Justin H. Nguyen, Takahisa Kanekiyo, Dennis W. Dickson, Sachiko Noda, Shigeto Sato, Nobutaka Hattori, Matthew S. Goldberg, Ian G. Ganley, Kelly L. Stauch, Fabienne C. Fiesel, Wolfdieter Springer

**Affiliations:** a Department of Neuroscience, Mayo Clinic,Jacksonville, FL, USA; b Department of Cardiovascular Medicine, Mayo Clinic,Jacksonville, FL, USA; c Division of Transplant Surgery, Department of Transplantation, Mayo Clinic,Jacksonville, FL, USA; d Neuroscience PhD Program, Mayo Clinic Graduate School of Biomedical Sciences, Jacksonville, FL, USA; e Department of Neurology, Juntendo University Graduate School of Medicine, Tokyo, Japan; f Center for Neurodegeneration and Experimental Therapeutics, Department of Neurology, University of Alabama at Birmingham, Birmingham, AL, USA; g MRC Protein Phosphorylation and Ubiquitylation Unit, School of Life Sciences, University of Dundee, Dundee, UK; h Department of Neurological Sciences, University of Nebraska Medical Center, Omaha, NE, USA

**Keywords:** aging, brain, heart, mice, mitochondria, mitophagy, phosphorylated ubiquitin, PINK1, PRKN, skeletal muscle

## Abstract

The ubiquitin kinase-ligase pair PINK1-PRKN identifies and selectively marks damaged mitochondria for elimination via the autophagy-lysosome system (mitophagy). While this cytoprotective pathway has been extensively studied *in vitro* upon acute and complete depolarization of mitochondria, the significance of PINK1-PRKN mitophagy *in vivo* is less well established. Here we used a novel approach to study PINK1-PRKN signaling in different energetically demanding tissues of mice during normal aging. We demonstrate a generally increased expression of both genes and enhanced enzymatic activity with aging across tissue types. Collectively our data suggest a distinct regulation of PINK1-PRKN signaling under basal conditions with the most pronounced activation and flux of the pathway in mouse heart compared to brain or skeletal muscle. Our biochemical analyses complement existing mitophagy reporter readouts and provide an important baseline assessment *in vivo*, setting the stage for further investigations of the PINK1-PRKN pathway during stress and in relevant disease conditions.

**Abbreviations:** ECL: electrochemiluminescence; ELISA: enzyme-linked immunosorbent assay; MSD: Meso Scale Discovery; PD: Parkinson disease; p-S65-Ub: Serine-65 phosphorylated ubiquitin; RT-PCR: real-time polymerase chain reaction; Ub: ubiquitin; WT: wild-type.

## Introduction

PINK1-PRKN directed mitophagy has emerged as a critical mitochondrial quality control pathway with likely far-reaching implications and relevance to stress, aging, and disease [1]. When mitochondria are damaged, the kinase PINK1 accumulates locally on the outer mitochondrial membrane where it phosphorylates ubiquitin (Ub) at serine-65 (p-S65-Ub) [2-4]. This signal acts as a specific receptor and allosteric activator of the E3 Ub ligase PRKN, which is recruited from the cytosol and structurally de-repressed [5-7]. PINK1 also phosphorylates a conserved serine-65 residue in the Ub-like domain of PRKN to fully activate and unleash the E3 Ub ligase [8-10]. PINK1 and PRKN then engage in a positive feedback loop to decorate damaged mitochondria with p-S65-Ub chains. These serve as a specific mitophagy label facilitating their autophagic delivery and elimination in lysosomes.

This process acts to maintain overall health and function of the mitochondrial network and thus is thought to be broadly cytoprotective. The importance of the pathway is further underscored as complete loss of PINK1 or PRKN function are the most common causes of early-onset Parkinson’s disease (PD) [1]. While PINK1-PRKN signaling seems particularly important for dopaminergic neurons that degenerate in PD [1,11,12], both enzymes are widely expressed in a variety of tissues and cell types. Alterations in PINK1-PRKN mitophagy are thought to play a role in many human age-related disorders including Alzheimer’s disease, cardiovascular disease, and myopathies and a prominent accumulation of p-S65-Ub is commonly seen in aging and disease [13-18].

PINK1 and PRKN have been extensively studied in cell culture often under overexpression conditions and upon acute and massive mitochondrial depolarization, but the relevance of PINK1-PRKN mitophagy is not well-established *in vivo* and debated [19]. In contrast to Drosophila, *pink1*^−/−^ or *prkn*^−/−^ mouse models only have subtle phenotypes and no change in overall mitophagy rates has been observed under basal conditions (reviewed in [20]). However, important *in vivo* roles for PINK1 and PRKN have been described in skeletal muscle and heart under physiological conditions and/or upon stress or injury [21-28]. Consistent with the idea of a broadly stress-activated pathway, various insults including infections, exhaustive exercise, advanced aging, and proteotoxic conditions all seem to increase PINK1-PRKN activity in mice or aggravate phenotypes in their absence [14,29-31].

Recently, we demonstrated that PINK1-PRKN signaling is indeed active in different human cell types and in rodent brain under basal conditions, albeit at extremely low levels thus requiring sensitive detection methods [32,33]. Here we expanded our analyses and determined the basal activity of the PINK1-PRKN pathway in brain, heart, and skeletal muscle of young, middle-aged, or old wild-type (WT) compared to *pink1*^−/−^ or *prkn*^−/−^ mice. Our results highlight similarities and differences between tissues with regards to the extent and regulation of PINK1-PRKN signaling during normal aging which contribute to a better understanding of this pathway on the organismal level.

## Results

### PINK1-PRKN signaling in brain, skeletal muscle, and heart of young mice

To determine PINK1-PRKN signaling in different organs, we first compared WT to age-matched *pink1*^−/−^ and *prkn*^−/−^ mice. Left mouse hemibrain (hereafter referred to as brain), mixed skeletal muscle (soleus and gastrocnemius; hereafter referred to as skeletal muscle), and entire hearts from young (3-month-old) animals were obtained and RNA and protein extracted. Quantitative real-time polymerase chain reaction (RT-PCR) confirmed the genotypes as no transcript of the corresponding genes was detectable in *pink1*^−/−^ and *prkn*^−/−^ mice. There were no differences in *Pink1 gene* expression between WT and *prkn*^−/−^ mice in any of the tissues (Fig. S1A). However, *Prkn* gene expression was somewhat reduced in all *pink1*^−/−^ tissues but only to a significant level in the brain (Fig. S1A-B).

**Figure 1.**
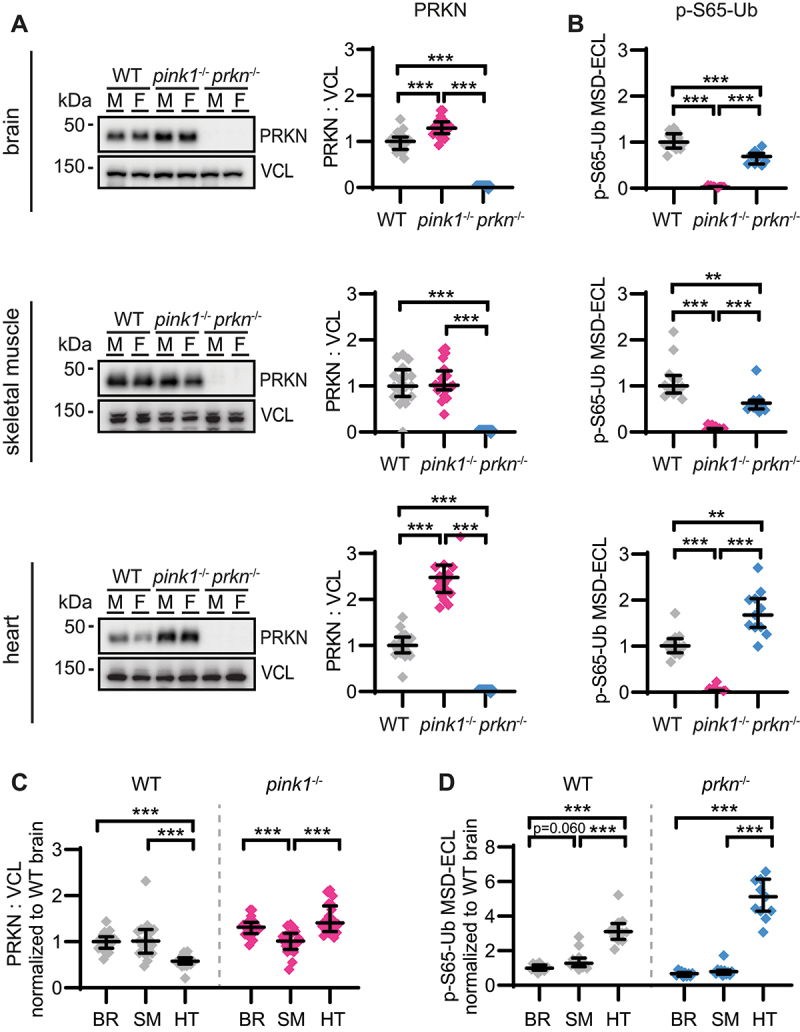
Basal PINK1-PRKN signaling in different tissue from young wild-type and *pink1*^−/−^ or *prkn*^−/−^ mice. PRKN and p-S65-Ub levels were measured in brain, skeletal muscle, and heart from 3-month-old WT, *pink1*^−/−^, and *prkn*^−/−^ mice by western blot or sandwich ELISA, respectively. (A) Representative western blots of PRKN with vinculin (VCL) as loading control are shown for the three organs (left panel) and their corresponding densitometric quantifications (right panel). M and F indicate male and female, respectively. To integrate the data from different blots and across different organs, the same two brain samples were loaded on each gel and used as calibrators after densitometric analysis. (B) MSD ELISA quantification of p-S65-Ub levels shown as electrochemiluminescence (ECL) signal in the three organs. Data is normalized with WT in the corresponding organs set to 1. (C) Quantification of PRKN levels in WT (gray dots) and *pink1*^−/−^ (pink dots) mice across brain (BR), skeletal muscle (SM), and heart (HT) after loading control (VCL) adjustment and calibration to the same two samples loaded on all gels with WT brain set to 1. (D) Quantification of p-S65-Ub levels across the same three organs within WT (gray dots) and *prkn*^−/−^ (blue dots) mice with WT brain set to 1. n=12 WT (6M/6F), 20 *pink1^−/−^* (10M/10F), and 11 *prkn^−/−^* (6M/5F). Data are shown as median ± interquartile range. Statistical analysis was performed using the Kruskal-Wallis test and then Mann-Whitney test followed by Bonferroni correction for multiple comparisons (*p<0.05, **p<0.01, ***p<0.001).

Due to lack of robust and reliable methods to measure mouse PINK1 protein, we next focused on PRKN and determined protein levels by western blot (Fig. 1A). PRKN protein was absent in different organs from *prkn*^−/−^ mice, as expected. We further observed a slight but significant increase (~1.3-fold) in PRKN protein in brain from *pink1*^−/−^ animals compared to WT, consistent with our recent report linking PINK1-mediated activation and turnover of PRKN [33]. While there was no such change noted in skeletal muscle at this young age, we observed an even greater increase in (inactive) PRKN protein (~2.5-fold) in *pink1*^−/−^ hearts pointing to higher activation and turnover of PRKN in this organ.

As a readout of the joint PINK1 Ub kinase and PRKN Ub ligase activity we measured p-S65-Ub levels using a Meso Scale Discovery (MSD) sandwich ELISA [32]. WT mice had significantly higher p-S65-Ub levels compared to the *pink1*^−/−^ animals, suggesting that the pathway is active under basal conditions in all three organs (Fig. 1B). Similar to previous results, p-S65-Ub levels in *prkn*^−/−^ mice compared to WT [33] were reduced by about 30% in brain and to a similar extent in skeletal muscle, reiterating that PRKN amplifies the formation of pS65-Ub in a positive feedback loop. Surprisingly, *prkn*^−/−^ hearts showed strongly elevated p-S65-Ub levels with almost 1.7-fold increase compared to hearts of age-matched WT mice, suggesting that different mechanisms are at play here.

Comparison of PRKN protein levels across the three organs in WT mice revealed similar levels between brain and skeletal muscle at 3-months-old, but much lower levels in heart reaching only 58% in comparison (Fig. 1C). Yet, in the absence of PINK1 at this age, PRKN protein levels were relatively similar in brain and heart, but lower in skeletal muscle of *pink1*^−/−^ mice. Comparison of p-S65-Ub across the organs in WT mice revealed slightly increased levels in skeletal muscle (~1.3-fold) compared to brain but much higher levels in heart (~3.1-fold) (Fig. 1D). In absence of PRKN, this difference in p-S65-Ub was even further pronounced rising to a more than 5-fold p-S65-Ub increase in heart from *prkn*^−/−^ mice compared to the other two organs. Except for slightly enhanced p-S65-Ub levels in *prkn*^−/−^ males compared to females, no other sex differences were observed for the levels of PRKN or p-S65-Ub (Fig. S1C-D).

Taken together, the combined analysis of mRNA, PRKN protein, and especially p-S65-Ub, revealed that the PINK1-PRKN pathway is active in all investigated organs, but to a different extent. Furthermore, data from the heart suggest a distinct regulation of PINK1-PRKN signaling that might be caused by differences in basal activation or an altered flux through the mitophagy pathway.

### Aging increases PINK1-PRKN signaling in brain, skeletal muscle, and heart of mice

We next expanded our analyses of PINK1-PRKN signaling in the three organs to an aging cohort consisting of young (3-month-old), middle-aged (13-month-old), and old (23-month-old) WT mice. Quantitative RT-PCR revealed that *Pink1* mRNA levels were significantly elevated with age in all three organs (1.3- to 1.5-fold) when comparing 3- and 23-month-old mice (Fig. S2A). *Prkn* mRNA levels generally increased with age across organs as well (1.2- to 1.6-fold) but were only statistically significant in brain tissue (Fig. S2B).

**Figure 2.**
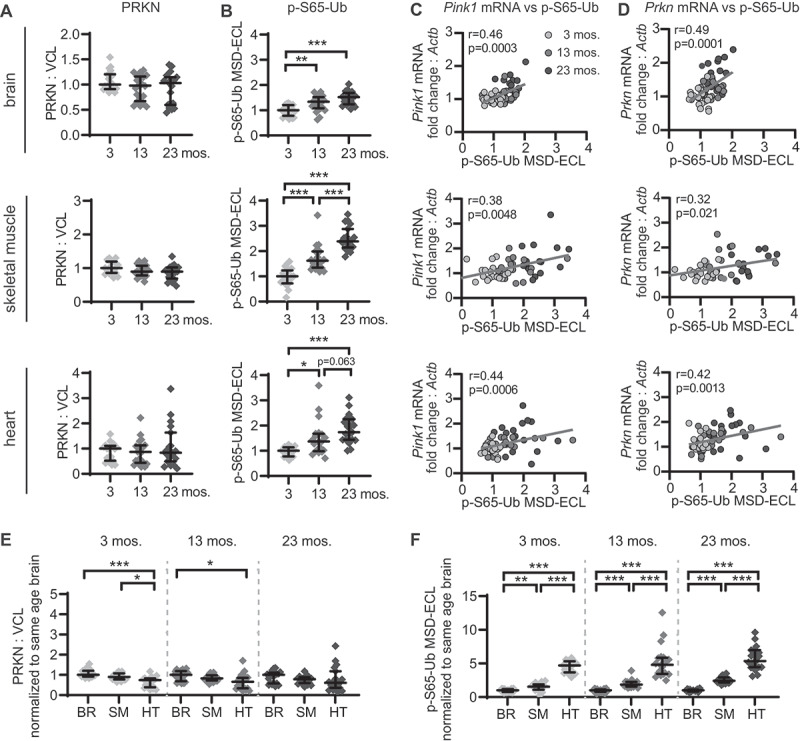
Age-dependent increase of p-S65-Ub and correlation with *Pink1* or *Prkn* expression in different mouse tissue in different mouse tissue. PRKN and p-S65-Ub levels were measured in brain, skeletal muscle, and heart from 3-, 13-, and 23-month-old WT mice by western blot or sandwich ELISA, respectively. (A) Densitometric quantification of PRKN protein levels in western blot relative the loading control VCL with WT brain set to 1 after calibration to the same two samples loaded on all gels. (B) MSD ELISA quantification of p-S65-Ub levels shown as ECL signal in the three organs during aging. Data is normalized with 3-month-old WT in the corresponding organ set to 1. (C, D) Spearman correlation of p-S65-Ub levels with *Pink1* mRNA (C), and *Prkn* mRNA (D) levels. (E, F) Comparison of PRKN (E) and p-S65-Ub (F) levels across brain (BR), skeletal muscle (SM), and heart (HT) within the same age groups with WT brain in each age group set to 1. n=19-20/age group (10M/10F). Data are shown as median ± interquartile range. Groupwise comparison was performed using the Kruskal-Wallis test and then Mann-Whitney test followed by Bonferroni correction for multiple comparisons (*p<0.05, **p<0.01, ***p<0.001).

In contrast to mRNA, PRKN protein levels did not alter with age in any of the analyzed tissue types (Fig. 2A). However, p-S65-Ub levels were significantly higher in older mice in all three organs with the largest increase in skeletal muscle (~2.4-fold), followed by heart (~1.7-fold) and brain (~1.5-fold) when comparing the youngest and oldest age groups (Fig. 2B). There was no significant correlation between mRNA and protein levels of PRKN in any of the tissues (Fig. S2C). Likewise, levels of PRKN protein and the mitophagy label p-S65-Ub did not correlate (Fig. S2D). Yet, gene expression of both *Pink1* and *Prkn* strongly correlated with the respective p-S65-Ub levels in all tissues across age (Fig. 2C-D). Relative to the brain, PRKN protein amounts were similar in skeletal muscle, but significantly lower in the heart at 3 and 13 months of age (Fig. 2E). In contrast, relative to the brain p-S65-Ub levels were greater in skeletal muscle and much higher in the heart at all ages (Fig. 2F).

We noted a few statistically significant sex differences during aging (Fig. S3). In young animals, levels of *Pink1* mRNA were elevated in skeletal muscle of males, while levels of p-S65-Ub were increased in brains of males compared to females. The most pronounced difference was observed in heart with females having much greater p-S65-Ub levels compared to males at mid age. However, even larger samples sizes may be needed to confirm these findings, and the biological significance remains to be established in each case.

In summary, during aging we identified increases in *Pink1* and *Prkn* mRNA as well as in p-S65-Ub levels across all tissues. PRKN protein levels however rather declined consistent with the notion that increased activation also facilitates the turnover of the E3 Ub ligase. In this context, it is noteworthy that compared to other tissues, heart showed the least amount of PRKN protein but the highest levels of p-S65-Ub.

### Aging intensifies tissue-specific PINK1-PRKN signaling

**Figure 3.**
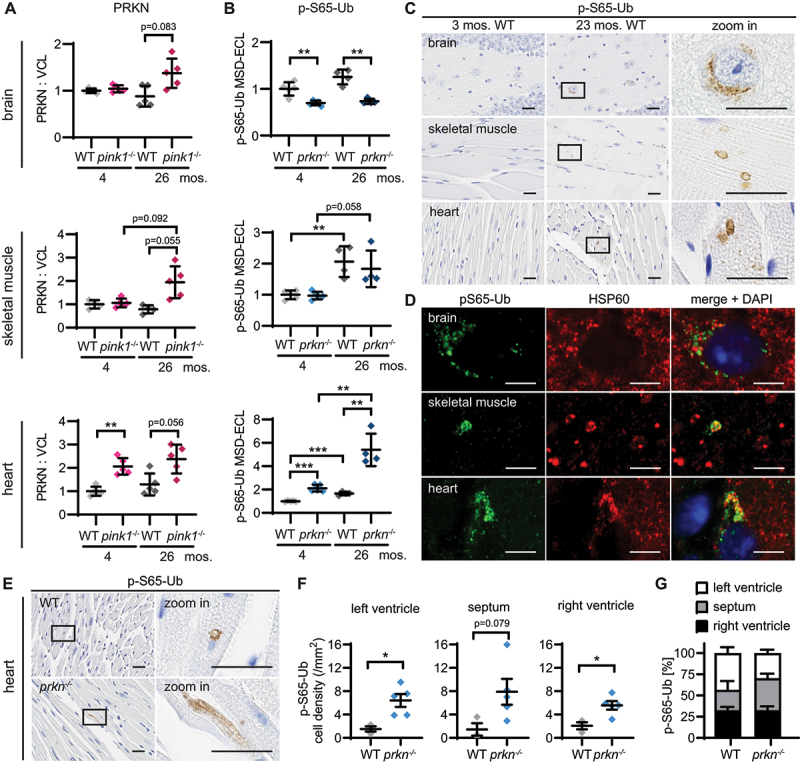
Age-dependent alterations of basal PINK1-PRKN signaling in organs from *pink1^−/−^* and *prkn^−/−^* mice. (A, B) Brain, skeletal muscle, and heart of young (4-month-old) and old (26-month-old) WT, *pink1*^−/−^ *or prkn^−/−^* mice were analyzed for levels of PRKN (A, for WT and *pink1^−^*^/-^) by western blot and of p-S65-Ub (B, for WT and *prkn*^−/−^) by sandwich ELISA. Each data is normalized to the 4-month-old WT values set to 1. n=5/genotype/age group. (C) Immunohistochemical assessment of p-S65-Ub levels in brain (representative images taken from the hippocampus), skeletal muscle (representative images taken from the gastrocnemius), and heart from 3- and 23-month-old WT mice. Zoom-in images are shown on the right. (D) Co-immunofluorescence staining of p-S65-Ub and mitochondrial marker HSP60 in brain, skeletal muscle, and heart from 23-month-old WT mice. (E) Immunohistochemical staining of p-S65-Ub in heart from 14-month-old WT and *prkn*^−/−^ mice. (F, G) p-S65-Ub positive cell density (F) and the distribution of positive cells (G) were manually quantified in three heart subregions, as indicated. n=3 for WT and n=5 for *prkn^−/−^* mice. Data are shown as mean ± SEM. Statistical analysis was performed using student t-test followed by Bonferroni correction for multiple comparisons (*p<0.05, **p<0.01, ***p<0.001). There was no statistical significance for the comparison between heart regions. Scale bar in immunohistochemical images: 12.5 µm. Scale bar in immunofluorescence images: 5 µm.

Next, we sought to further evaluate findings in *pink1*^−/−^ and *prkn*^−/−^ animals at later ages and using independent methods. Therefore, we utilized different sets of WT and *pink1*^−/−^ or *prkn*^−/−^ mice with young (4-month-old) or advanced age (26-month-old). Consistent with the results from the larger aging cohort (Fig. 2), there was no significant change in PRKN levels in WT animals over age (Fig. 3A). The increase of PRKN protein in young *pink1*^−/−^ mice was weaker and statistical significance was lost for brain in this smaller cohort (Fig. 3A), compared to Fig. 1. However, PRKN levels were increased 1.6- to 2.6-fold in brain and in skeletal muscle of old *pink1*^−/−^ compared to age-matched WT animals, respectively. In the heart, PRKN levels were significantly increased in young *pink1*^−/−^ mice compared to young WT animals and the fold change between the two genotypes remained similar with age. p-S65-Ub levels increased over age in all tissue types (Fig. 3B), although the significance for brain, which showed the smallest fold change of all organs in the larger cohort (Fig. 2), was lost. In young *prkn*^−/−^ mice, p-S65-Ub levels were decreased in brain, but not in skeletal muscle compared to age-matched WT mice (Fig. 3B), consistent with Fig. 1. There was no further change with age in either tissue type. In heart, p-S65-Ub levels were again strongly increased in young *prkn*^−/−^ mice (2.1-fold) and further elevated in old *prkn*^−/−^ animals compared to age-matched WT (3.3-fold).

To visualize the results, we examined p-S65-Ub by staining paraffin sections of brain, skeletal muscle, and heart from young and old WT mice. Tissues from *pink1*^−/−^ animals that were used as negative control were devoid of p-S65-Ub signal (data not shown). In young WT mice, p-S65-Ub immunoreactivity was very faint in all three tissues. Tissue from older WT mice, however, showed overall more frequent and more intense p-S65-Ub signal consistent with the biochemical results (Fig. 3C). Super-resolution fluorescence microscopy (Airyscan) confirmed that the p-S65-Ub signal in older WT mice either strongly co-localized or at least was adjacent to the mitochondrial matrix-localized HSP60 signal across all three tissues (Fig. 3D). To further investigate the elevated levels of p-S65-Ub observed in heart upon loss of the E3 Ub ligase PRKN, we stained sections and compared p-S65-Ub levels amongst age-matched 14-month-old WT and *prkn*^−/−^ mice (Fig. 3E). Indeed, staining independently showed a significant increase of p-S65-Ub signal in hearts from *prkn*^−/−^ compared to WT controls with an overall 3.7-fold change (Fig. 3F), comparable to the 2.1- or 3.3-fold increase measured by MSD in the 4- and 26-month-old cohort, respectively. This signal was increased in both the left and right ventricle with no obvious change in p-S65-Ub distribution between these different compartments when comparing WT and *prkn*^−/−^ mice (Fig. 3G).

Taken together, our results confirm and moreover highlight both similarities and distinctions with regard to PRKN protein and p-S65-Ub levels in different tissues with advanced age. Normal aging seems to amplify some of the genotype-specific effects in mitophagy further suggesting distinct activation and turnover rates between different tissues, with heart being the most affected.

## Discussion

PINK1-PRKN mitophagy has been recognized as an important mitochondrial quality control pathway, but this has been extremely difficult to ascertain *in vivo* in mice. Using a biochemical approach, we have previously shown that PINK1-PRKN signaling is active in mouse brain even at baseline [32,33]. Here we extended our prior study and compared brain to other energetically demanding tissues such as skeletal muscle and heart in young animals and during aging. Combined analyses of gene expression, protein levels, and enzymatic activities revealed genotype-specific and age-dependent differences regarding the regulation and extent of PINK1-PRKN signaling in these organs. The current assessment of activities and altered fluxes at baseline provides a framework for further and comparative investigations of the PINK1-PRKN pathway under various stress and disease relevant conditions.

In line with previously described roles under physiological and pathological conditions [21-26,32,33], our data demonstrated that the PINK1-PRKN pathway is broadly active in mice *in vivo*, even at young age. Brain, skeletal muscle, and heart from WT mice all had significantly higher p-S65-Ub levels compared to the same organ from *pink1*^−/−^ mice. In young mice, p-S65-Ub levels in heart were about 3- to 5-fold higher, while levels in skeletal muscle were about 1.3- to 1.5-fold higher than in brain. The widespread basal activity of the pathway is in line with a generally broad expression of *PINK1* and *PRKN* in different cell and tissue types. Consistent with a general age-dependent increase of p-S65-Ub seen in human autopsy brain [13,17], p-S65-Ub levels also increased with aging in mouse tissues (from 3 to 23 months) with the least increase seen in brain (1.5-fold), followed by heart (1.7-fold), and the most increase seen in skeletal muscle (2.4-fold). In aged animals the difference in PINK1-PRKN signaling between the different organs was even more pronounced. At 23 months, heart and skeletal muscle had 5.4-fold and 2.4-fold higher p-S65-Ub levels compared to brain, respectively. The generation of p-S65-Ub is primarily driven by mitochondrial damage, but a decline in autophagic-lysosomal degradation could also contribute to its build-up. Here, we found a general increase of both *Pink1* and *Prkn* mRNA with aging in all three organs, suggesting age-dependent gene expression changes may also contribute to increased PINK1-PRKN signaling.

Recently, we described that endogenous PRKN protein accumulates in the absence of PINK1, indicating that its activation and turnover are tightly linked [33]. Consistently, PRKN levels were slightly but significantly increased in young *pink1^−/−^* brain here as well (1.3-fold) at least in the larger cohort. While we did not observe a statistically significant effect at 3-4 months of age, another recent study reported increased PRKN protein levels in skeletal muscle of 6-month-old *pink1^−/−^* animals [34]. The most robust effect was observed in the heart though. Here, loss of PINK1 resulted in an almost 2.5-fold increase in PRKN protein. WT hearts displayed the greatest levels of p-S65-Ub but had the lowest PRKN protein relative to the other organs. Together this suggests that basal PINK1-PRKN signaling is most active in the heart and this may also be reflected by the cardiac phenotypes of knockout mice that appear most prominent under stress or disease conditions [20,21,24,26-28]. *Prkn* mRNA levels however were rather decreased in *pink1^−/−^* tissue potentially as an adaptive response to accumulation of inactive PRKN protein. The respective fold increase in PRKN protein seen in *pink1*^−/−^ tissue seemed even more pronounced with age. Moreover, in WT animals, PRKN protein levels rather declined with age as the enzyme may be increasingly activated and “consumed” by mitophagy [33]. However, more animals are certainly needed to substantiate both findings. Collectively, our data suggest the changes observed in relative PRKN protein levels in WT or *pink1*^−/−^ animals might be used as surrogate for distinct basal mitophagy rates across the different tissues and ages.

The E3 Ub ligase PRKN is known to engage in a positive feedback loop together with the Ub kinase PINK1 during which PRKN provides more substrates for PINK1 [35-37]. Accordingly, in brain and skeletal muscle from *prkn*^−/−^ animals, the levels of the joint product p-S65-Ub were reduced by more than 1.5-fold. This is similar to what is seen with PRKN-deficient cell cultures in which the pathway has been maximally activated by chemical depolarization of mitochondria [33]. In stark contrast however, p-S65-Ub levels were 1.6-fold increased in *prkn*^−/−^ hearts, pointing towards a mechanistic difference in heart vs the other organs. This effect was even further pronounced in hearts from older *prkn*^−/−^ animals. The heart-specific increase of p-S65-Ub in *prkn*^−/−^ was independently confirmed by immunohistochemistry and was detected rather broadly across heart subregions. The elevated p-S65-Ub levels were not a result of increased *Pink1* expression as transcript levels did not change in absence of PRKN. While the underlying mechanisms remain uncertain, mechanistic explanations include other potential compensatory changes amongst enzymes that may affect de-/conjugation of p-S65-Ub or a block in degradation as suggested in PRKN deficient flies, which also present with increased p-S65-Ub levels [38].

Although the described biochemical assessment of p-S65-Ub is highly sensitive and selective for PINK1-PRKN signaling, our study has certain limitations and thus findings have to be interpreted with caution. We have only been able to quantify protein levels of the E3 Ub ligase PRKN, but not of the Ub kinase PINK1 given to the current inability of the field to reliably detect the mouse protein. While elevated levels of p-S65-Ub and concomitantly decreased levels of PRKN protein are indicative of increased mitophagy with age, we did not determine actual mitophagy rates here. We also did not measure mitochondrial mass, morphology, or function, which may provide additional important context. Yet, it should be noted that there is a well described general decline in mitochondrial structure and function with age [34,39,40]. In contrast to the earlier described notion of a widespread decline in mitophagy *in vivo* with age [41], two recent studies report basal mitophagy to remain stable or even increase throughout the lifespan of mice [42,43]. Using a fluorescent mitophagy reporter, they provide a refined spatiotemporal analysis of basal mitophagy highlighting marked regional and cell type dependent fluctuations during aging. While such reporters measure all mitophagy pathways collectively [44-47], pronounced differences in mitophagy especially in the heart [21,26,28] but also in skeletal muscle [22,34] have been described when comparing WT and *pink1^−/−^* or *prkn*^−/−^ mice under stress conditions.

In light of these new findings [42,43] and given that we only analyzed hemibrains, whole hearts, and mixed skeletal muscles, it will be important to better resolve our findings across brain regions, muscle fibers, and individual cell types. Beyond the organs studied herein, there was a general increase in p-S65-Ub in other mouse tissue during aging too (data not shown), but there was also more variability noted especially with more advanced age. While some of this is likely due to different organ-specific matrix effects, it is important to note that PINK1-PRKN signaling is a dynamic pathway that cycles between activation and degradation. Thus, some variation can be expected *in vivo* at any time and this might be further influenced by stress due to housing conditions, caloric intake, activity, and/or infections. Overall, even larger samples sizes may be needed, especially at older age, to fully validate suggestive sex differences. In this context it will be important to determine whether one sex is driving the observed effects in presence or absence of PINK1 or PRKN.

Altogether, our work demonstrates that PINK1-PRKN signaling is constitutively active *in vivo* in mouse. In brain, skeletal muscle, and heart, we found significant differences in regulation and basal activity of the PINK1-PRKN pathway during normal aging. Our analysis complements existing broad mitophagy reporter readouts and sets the stage for follow-up analyses during mtDNA mutagenic stress or exhaustive exercise, and in the context of other genetic or pharmacological challenges known to further activate PINK1-PRKN signaling.

## Materials and methods

### Mice

All mouse procedures were performed in accordance with the Mayo Clinic Institutional Animal Care and Use Committee policies. *pink1*^−/−^ and *prkn*^−/−^ mice (Jackson Laboratories, strains 017946 and 006582, respectively) [48,49] were backcrossed over 3 generations to a C57BL/6J background (Jackson Laboratories, 00664). The final cohort to study genotype differences consisted of 3-month-old ± 2 weeks WT (n=12, 6 males and 6 females), *pink1*^−/−^ (n=20, 10 males and 10 females), and *prkn*^−/−^ mice (n=11, 6 males and 5 females). A cohort of WT mice (C57BL/6J) of different ages was received from the National Institute on Aging (NIA). The cohort consisted of 59 mice: 3-month-old ± 2 weeks (n=20, 10 males and 10 females), 13-month-old ± 2 weeks (n=20, 10 males and 10 females) and 23-month-old ± 2 weeks (n=19, 10 males and 9 females).

Additional cohorts were used to study genotype effects during aging and for further validation. For WT and *pink1*^−/−^ the cohort consisted of 20 mice: young (4-month-old) WT (n=5, 3 males and 2 females) and *pink1*^−/−^ (n=5, 3 male and 2 females), and old (26-month-old) WT (n=5, 3 males and 2 females) and *pink1*^−/−^ (n=5, 3 male and 2 females). For WT and *prkn*^−/−^, we received tissue from Dr. Nobutaka Hattori (Juntendo University, Japan) [50]. The tissue was harvested from young (4-month-old ± 2 weeks) WT (n=5, all males), and *prkn*^−/−^ (n=5, all males), and old (26-month-old ± 2 weeks) WT (n=4, all males) and *prkn*^−/−^ animals (n=4, all males). For imaging analysis, two young (3-month-old, 1 male and 1 female) and old (23-month-old, 1 male and 1 female) WT mice were used for immunohistochemical staining across different organs. Tissues from two *pink1*^−/−^ mice (14-month-old, 1 male and 1 female) were used as negative control. Additionally, a group of 14-month-old ± 2 weeks WT (n=3, all males) and *prkn*^−/−^ (n=5, 3 males and 2 females) was used for immunohistochemical analysis in heart.

### Mouse tissue

All mice were anesthetized and perfused intracardially with cold PBS (Thermo Fisher Scientific, 14190235). Left brain hemisphere (referred to as brain), mixed muscle (combined soleus and gastrocnemius; referred to as skeletal muscle), and entire hearts were harvested from each mouse and immediately flash frozen using liquid nitrogen. Frozen tissues were stored at -80 °C and remained frozen on dry ice during weighing. Mouse tissues were homogenized using 2 mL glass homogenizers (Fisher Scientific, K885300-0002) in 5x volumes of ice-cold TBS buffer (50 mm Tris pH 7.4 [Thermo Scientific, J75825-A4], 150 mm NaCl [Fisher Scientific, BP358-10]) containing phosphatase and protease inhibitors (Roche, 04906845001, 05056489001) (TBS+). Both plunger A and then B, were used for 15 strokes each and TBS+ homogenates aliquoted and flash frozen in liquid nitrogen until further extraction.

### RNA extraction and quantitative RT-PCR

A total of 50 µL TBS homogenate from mouse brain, skeletal muscle, and heart was used for RNA extraction using a RNeasy Plus Mini Kit (Qiagen, 74134). Quantitative RT-PCR was carried out using iTaq Universal SYBR Green One-Step Kit (Bio-Rad, #1725150). 50 ng of RNA was mixed with primers for targeted genes, SYBR Green, and iScript reverse transcriptase in a 5 µL reaction. The PCR was executed using a 384-well block on a LightCycler 480 system (Roche, Switzerland). Relative transcript levels for *Pink1* and *Prkn* were calculated with 2-^ΔΔCT^ using *Actb* as housekeeping gene and normalized to the relative expression level of the corresponding tissue from 3-month-old mice. The following primers were used: *Pink1* forward 5’-GAGTGGGACTCAGATGGCTGTCC-3’, *Pink1* reverse 5’-CCAGAATGGGCTGTGGACACCTC-3’, *Prkn* forward 5’- GATTCAGAAGCAGCCAGAGG-3’, *Prkn* reverse 5’-GGTGCCACACTGAACTCG-3’, *Actb* forward 5’-AGTGTGACGTTGACATCCGTA-3’, *Actb* reverse 5’-GCCAGAGCAGTAATCTCCTTC-3’.

### Protein extraction

Tissue lysis and protein extraction was completed by adding 25 µL of 5x RIPA buffer (50 mm Tris, pH 8.0 [Thermo Scientific, J75825-A4], 150 mm NaCl [Fisher Scientific, BP358-10], 0.1% SDS [Fisher Bioreagents, BP166-500], 0.5% deoxycholate [Sigma, D6750], 1% NP-40 [United States Biochemical, 19628]) to 100 µL TBS homogenate, vortexed and incubated for 30 minutes on ice. During the tissue homogenization and lysing steps, homogenates and lysates were always placed on ice. After incubation, lysates were centrifuged twice at 20,000 x *g* for 15 minutes at 4 °C to remove insoluble components, lipids, and nucleic acids. The resulting supernatant was transferred to a fresh tube each time. Protein concentration was determined using a Bicinchoninic acid assay (ThermoFisher, 23225).

### Western blot

Protein lysates were prepared in Laemmli buffer (62.5 mm Tris HCl pH 6.8, 1.5% SDS [w:v], 8.33% glycerol [v:v] [Fisher Scientific, BP229-1], 1.5% β-mercaptoethanol [v:v] [Sigma, M3148], 0.005% bromophenol blue [w:v] [Sigma, 5525]) and boiled at 95°C for five minutes. Protein electrophoresis was performed using 8-16% Tris-Glycine gels (Invitrogen, XP08165BOX) and standard running buffer (25 mm Tris, 0.2 M Glycine, 0.1% SDS) at room temperature. 30 µg of total protein was loaded per well. For comparison between blots and across organs, the same two mouse tissue lysate samples were used as calibrators and loaded onto each gel for normalization of the signals after densitometric analysis. Proteins were transferred onto polyvinylidene fluoride (PDVF) membranes (Millipore, IEVH00005). Blocking was performed with 5% non-fat milk dissolved in TBST for 1 hour followed by incubation with primary antibodies PRKN (Cell Signaling Technology, 2132) at 1:2000 dilution in 5% BSA in TBST at 4 °C overnight and VCL (Sigma-Aldrich, V9131) as the loading control at 1:375,000 dilution in 5% milk for 1 hour at room temperature. Next, the membranes were washed for 30 minutes in TBST and incubated with the secondary HRP-conjugated antibody (Jackson Immuno research, 111-035-003 [anti-rabbit], 715-035-150 [anti-mouse]) in 5% milk for 1 hour at room temperature. All western blots were generated under identical conditions (amount of protein loaded, gel/membrane type, antibodies and concentrations) and analyzed with identical settings and exposure times to ensure comparability. The signal was developed using an HRP Substrate (Millipore, WBKLS0500) and imaged using a ChemiDoc MP imaging system (Bio-Rad, Hercules, CA, USA) with identical settings and exposure times. Western blot signal intensity was determined using the ImageStudio Lite software (version 5.2). After densitometric analysis, PRKN levels were adjusted by their VCL loading control and then normalized to the blot calibrators.

### P-S65-Ub Meso-Scale Discovery sandwich ELISA

Our assay to detect p-S65-Ub has been described before [32]. Briefly, the 96-well MSD plates (Meso Scale Diagnostics, L15XA) were coated with 1 µg/mL p-S65-Ub capturing antibody (Cell Signaling Technology, 62802), blocked with 1% BSA in TBST. Then 30 µg/30 µl protein lysates were added to each well and after 2 h incubation, 5 µg/mL ubiquitin antibody (Thermo Fisher Scientific, 14-6078-37) was used for detection followed by a sulfo-tag coupled secondary antibody (Meso Scale Diagnostics, R32AC-1). The plates were then read on a MESO QuickPlex SQ 120 reader (Meso Scale Diagnostics, Rockville, MD, USA). All samples were run in duplicates and each plate had an organ specific *pink1*^−/−^ sample included as a negative control as well as a recombinant K48 p-S65-Ub tetramer (Boston Biochem, UC-250) as a positive control. The raw values were background corrected by subtracting the organ-specific *pink1^−/−^* value.

### Immunohistochemistry

Immunohistochemical staining was performed with paraffin-embedded right sagittal hemibrain (referred to as brain), skeletal muscle (combined soleus and gastrocnemius; referred to as skeletal muscle), and ventricular heart sections (one section per organ for each animal). Sections were cut at a thickness of 5 microns and mounted onto positively charged slides to dry overnight at 60°C. After sections were deparaffinized and rehydrated, antigen retrieval was performed by steaming the sections for 30 minutes in deionized water. Immunostaining was carried out using Envision Plus kit (Agilent, K4011). Endogenous peroxidase blocking was performed for 5 minutes using hydrogen peroxide (Swan, L0011380FB). Following blocking with 5% normal goat serum (Invitrogen, 16210-072) for 20 minutes, sections were incubated with primary antibody against pS65-Ub (in-house, 1:250) [17] that was diluted in Dako Antibody Diluent with Background Reducing Components (Agilent, S3022) for 45 minutes at room temperature. Subsequently, sections were incubated with EnVision-Plus anti-rabbit labeled polymer-HRP (Agilent, K4003) for 30 minutes at room temperature. Peroxidase labeling was visualized with the chromogen solution 3,3’-diaminobenzidine (Agilent, K3468). Sections were then counterstained with Gill 1 hematoxylin (Epredia, 6765006) and following dehydration, coverslipped with Cytoseal XYL mounting media (Epredia, 8312-4). After drying, sections were scanned with an Aperio AT2 digital pathology scanner (Leica Biosystems, Wetzlar, Germany) at 20x magnification and additionally acquired at 100x magnification brightfield using BZ-X810 All-in-One Fluorescence Microscope (Keyence, Osaka, Japan).

### Immunofluorescence

Following target retrieval and blocking (same as the immunohistochemical staining), brain, skeletal muscle, and heart paraffin embedded sections were blocked (Agilent, X090930-2) for 1 hour at room temperature before staining with primary antibodies targeting p-S65-Ub (in house, 1:250) and HSP60 (Arigo, ARG10757, 1:300) overnight at 4°C in a humidified chamber with a blocking reagent (Agilent, S302281-2). The following day, sections were washed with PBS prior to blocking with secondary antibodies (ThermoFisher, A-11008 and A-21449, 1:500) with DAPI (Sigma-Aldrich, D9542, 1:1000) for 1.5 hours at room temperature. Autofluorescence was quenched for 30 seconds using TrueBlack (Biotium, 23007) before mounting with a #1.5 thickness coverslip in fluorescence mounting medium (Agilent, S302380). Super-resolution confocal (Airyscan) images were taken with a LSM 880 microscope (Zeiss, Oberkochen, Germany) with z-stack.

### Statistical analysis

Data were analyzed using GraphPad Prism (version 9.2.). Given that most measures were not normally distributed and had differing variances between groups, non-parametric tests (Kruskal-Wallis and Mann-Whitney U tests followed by adjustment with Bonferroni correction) were used. For groups with sample size less than 6, a parametric test (Student’s t-test followed by adjustment with Bonferroni correction) was used. Correlation analysis was performed using Spearman’s test. For Bonferroni correction, all tests were corrected for three organs and additionally for multiple pair-wise comparisons between age, genotype, and/or sex groups.

## Supplementary Material

Supplementary material_aging mice_2nd revison.docx

## Data Availability

Access to the datasets used and/or analyzed during the current study are available from the corresponding author Dr. Wolfdieter Springer (Springer.Wolfdieter@mayo.edu) upon request.
