# Supplementary material for: Alterations of PINK1-PRKN signaling in mice during normal aging
Source: Autophagy Rep. 2024 Dec 7;3(1):2434379. doi: 10.1080/27694127.2024.2434379 (PMC11855339; doi:10.1080/27694127.2024.2434379)
Supplement: Supplementary material_aging mice_2nd revison.docx [file KAUO_A_2434379_SM7727.docx]

# SUPPLEMENTARY MATERIAL

# Alterations of PINK1-PRKN signaling in mice during normal aging

Zahra Baninameh^1,#^, Jens O. Watzlawik^1,#^, Xu Hou^1,#^, Tyrique Richardson^1^, Nicholas W. Kurchaba^1^, Tingxiang Yan^1^, Damian N. Di Florio^1^, DeLisa Fairweather^2^, Lu Kang^3^, Justin H. Nguyen^3^, Takahisa Kanekiyo^1,4^, Dennis W. Dickson^1,4^, Sachiko Noda^5^, Shigeto Sato^5^, Nobutaka Hattori^5^, Matthew S. Goldberg^6^, Ian G. Ganley^7^, Kelly L. Stauch^8^, Fabienne C. Fiesel^1,4^ and Wolfdieter Springer^1,4, *^

^1^ Department of Neuroscience, Mayo Clinic, Jacksonville, FL 32224, USA

2 Department of Cardiovascular Medicine, Mayo Clinic, Jacksonville, FL 32224, USA

3 Division of Transplant Surgery, Department of Transplantation, Mayo Clinic, Jacksonville, FL 32224, USA

4 Neuroscience PhD Program, Mayo Clinic Graduate School of Biomedical Sciences, Jacksonville, FL 32224, USA

^5^ Department of Neurology, Juntendo University Graduate School of Medicine, Tokyo 113-8421, Japan

^6^ Center for Neurodegeneration and Experimental Therapeutics, Department of Neurology, University of Alabama at Birmingham, Birmingham, AL 35233, USA

^7^ MRC Protein Phosphorylation and Ubiquitylation Unit, School of Life Sciences, University of Dundee, Dundee DD1 5EH, UK

^8^ Department of Neurological Sciences, University of Nebraska Medical Center, Omaha, NE 68198

^#^ contributed equally

*correspondence should be addressed to:

Wolfdieter Springer, PhD, Department of Neuroscience, Mayo Clinic, 4500 San Pablo Road, Jacksonville, FL 32224, USA. Tel.: +1-904-953-6129; Fax: +1-904-953-7117; Email: [Springer.Wolfdieter@mayo.edu](mailto:Springer.Wolfdieter@mayo.edu)

**
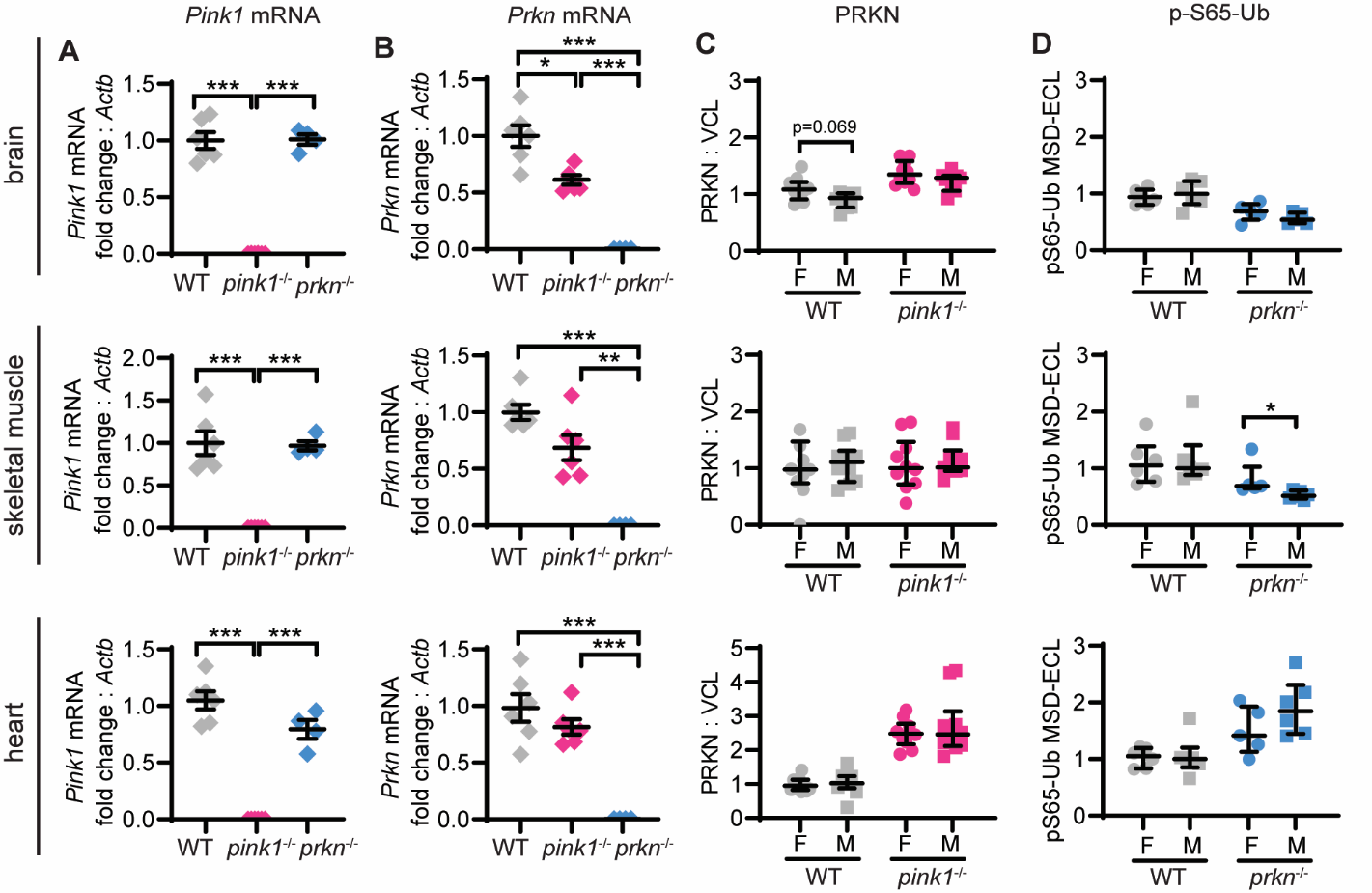
**

**Supplementary Figure 1. Gene expression and sex differences in basal PINK1-PRKN signaling in young *pink1^-/-^* and *prkn^-/-^* mice.** (**A**, **B**) *Pink1* and *Prkn* mRNA levels were measured in brain, skeletal muscle, and heart from 3-month-old wildtype (WT), *pink1^-/-^,* and *prkn^-/-^* mice by real-time qPCR. *Actin* was used as housekeeping gene. *Pink1* (**A**) and *Prkn* (**B**) mRNA levels were compared between genotypes in each tissue. Data is normalized with WT in the corresponding organs set to 1 and shown as mean ± SEM. n=6 WT (3M/3F), 6 *pink1^-/-^* (3M/3F), 4 *prkn^-/-^* (1M/3F). Statistical analysis was performed using a one-way ANOVA followed by Bonferroni correction for multiple comparisons (*p<0.05, **p<0.01, ***p<0.001). (**C, D**) PRKN and p-S65-Ub protein levels were measured in the same tissue by western blot (for PRKN) or sandwich ELISA (for p-S65-Ub) and were compared between female and male mice within each genotype. (**C**) Densitometric quantifications of PRKN protein levels in western blot relative the loading control VCL. (**D**) MSD ELISA quantification of p-S65-Ub levels shown as electrochemiluminescence (ECL) signal. Data is normalized with WT in the corresponding organs set to 1 and shown as median ± interquartile range. n=12 WT (6M/6F), 20 *pink1^-/-^* (10M/10F), and 11 *prkn^-/-^* (6M/5F). Statistical analysis was performed using the Mann-Whitney test followed by Bonferroni correction for multiple comparisons (*p<0.05).

**
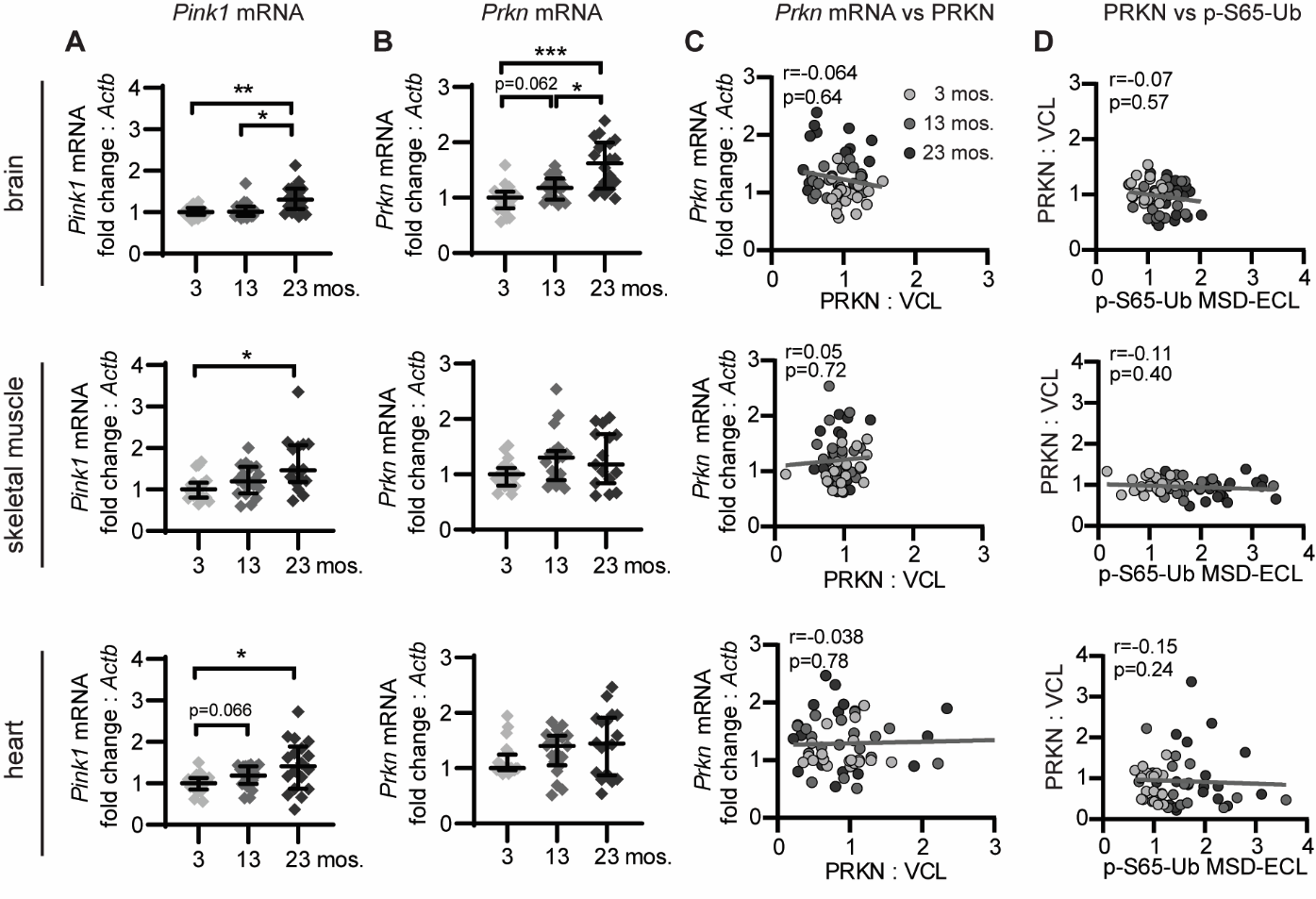
Supplementary Figure 2. Gene expression and correlation analysis in basal PINK1-PRKN signaling in mice during aging.** (**A, B**) *Pink1* and *Prkn* mRNA levels were measured in brain, skeletal muscle, and heart from 3-, 13-, and 23-month-old WT mice by realtime qPCR. *Actin* was used as housekeeping gene. *Pink1* (**A**) and *Prkn* (**B**) mRNA levels were compared during aging in each tissue. Data is normalized with 3-month-old WT in the corresponding organ set to 1 and shown as median ± interquartile range. (**C, D**) Correlation of PRKN protein levels with *Prkn* mRNA (**C**), and p-S65-Ub (**D**) levels in the same cohort. n=19-20/age group (10M/10F). Groupwise comparison was performed using Mann-Whitney test followed by Bonferroni correction for multiple comparisons (*p<0.05, **p<0.01, ***p<0.001). Correlation analysis was performed using Spearman’s test.

**
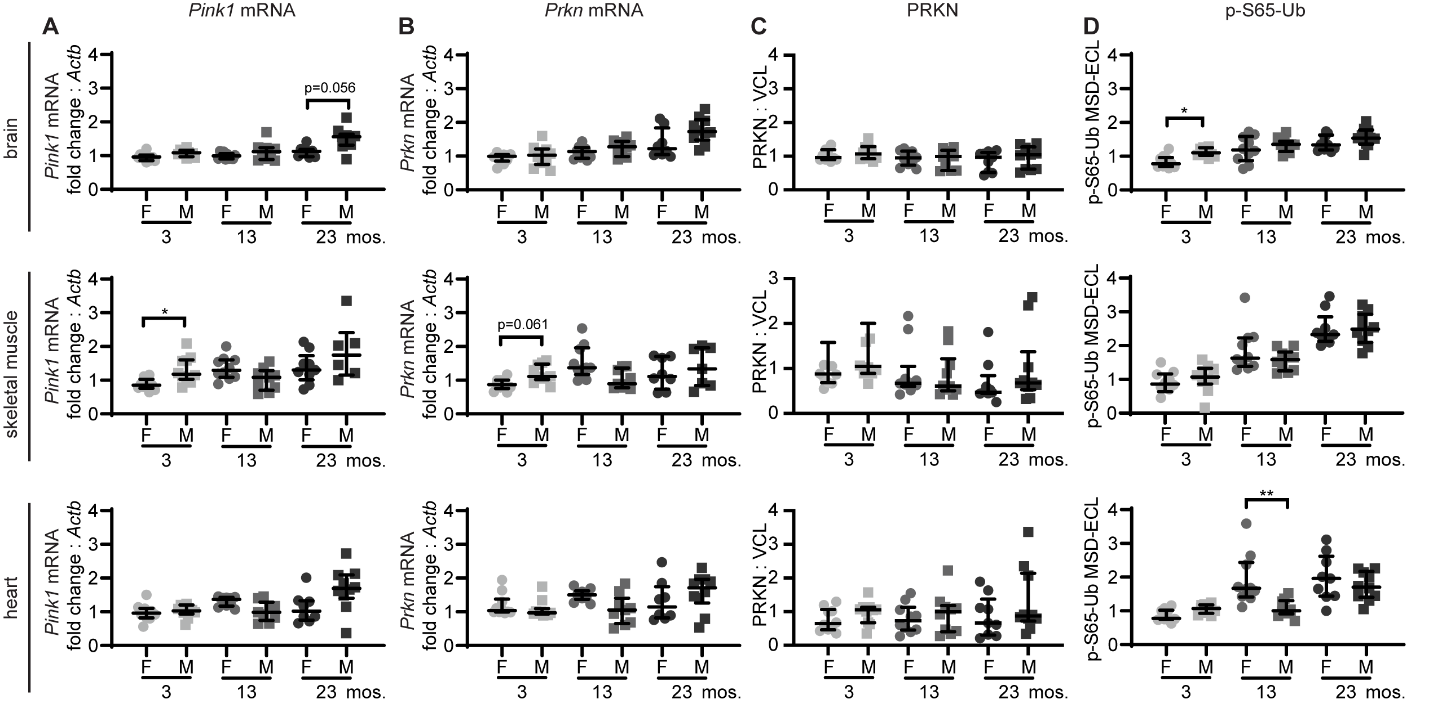
**

**Supplementary Figure 3. Sex differences in basal PINK1-PRKN signaling in mice during aging.** (**A**, **B**) *Pink1* and *Prkn* mRNA levels were measured in brain, skeletal muscle, and heart from 3-, 13-, and 23-month-old WT mice by realtime qPCR. *Actin* was used as housekeeping gene. *Pink1* (**A**) and *Prkn* (**B**) mRNA levels were compared between female and male mice within each age group. (**C, D**) PRKN and p-S65-Ub protein levels were measured in the same tissue by western blot (for PRKN) or sandwich ELISA (for p-S65-Ub) and were compared between female and male mice within each age group. (**C**) Densitometric quantifications of PRKN protein levels in western blot relative the loading control VCL. (**D**) MSD ELISA quantification of p-S65-Ub levels shown as electrochemiluminescence (ECL) signal. Data is normalized with 3-month-old WT in the corresponding organs set to 1 and shown as median ± interquartile range. n=19-20/age group (10M/10F). Statistical analysis was performed using the Mann-Whitney test followed by Bonferroni correction for multiple comparisons (*p<0.05, **p<0.01).
